# A Rapid and Convenient Approach to Construct Porous Collagen Membranes *via* Bioskiving and Sonication-Feasible for Mineralization to Induce Bone Regeneration

**DOI:** 10.3389/fbioe.2021.752506

**Published:** 2021-10-11

**Authors:** Zhenzhen Wu, Juan Zhong, Yingjie Yu, Mingdeng Rong, Tao Yang

**Affiliations:** ^1^ Department of Periodontology and Implantology, Stomatological Hospital, Southern Medical University, Guangzhou, China; ^2^ Hospital of Stomatology, Guangdong Provincial Key Laboratory of Stomatology, Institute of Stomatological Research, Guanghua School of Stomatology, Sun Yat-sen University, Guangzhou, China; ^3^ Department of Biomedical Engineering, Tufts University, Boston, MA, United States

**Keywords:** porous collagen membrane, bioskiving, sonication, mineralization, bone regeneration

## Abstract

Porous mineralized collagen membranes efficiently promote bone regeneration. To generate them, we need to fabricate collagen membranes that are porous. However, the current fabrication method is primarily based on a bottom-up strategy, with certain limitations, such as a long manufacturing process, collagen denaturation, and failure to control fibril orientation. Using a top-down approach, we explore a novel method for constructing porous collagen membranes *via* the combined application of bioskiving and sonication. Numerous collagen membranes with well-aligned fibril structures were rapidly fabricated by bioskiving and then sonicated at 30, 60, 90, and 120 W for 20 min. This treatment allowed us to study the effect of power intensity on the physicochemical traits of collagen membranes. Subsequently, the prepared collagen membranes were immersed in amorphous calcium phosphate to evaluate the feasibility of mineralization. Additionally, the bioactivities of the membranes were assessed using preosteoblast cells. Tuning the power intensity was shown to modulate fibril orientation, and the porous membrane without denatured collagen could be obtained by a 20-min sonication treatment at 90 W. The prepared collagen membrane could also be further mineralized to enhance osteogenesis. Overall, this study offers a rapid and convenient approach for fabricating porous collagen membranes *via* bioskiving and sonication.

## Introduction

The clinical treatment of alveolar bone defects caused by periodontitis and trauma has advanced considerably with the use of the guided bone regeneration (GBR) technique. Despite various issues associated with the GBR technique, the GBR membrane has a crucial function (Turri et al., 2016; Wu et al., 2020; Farnezi Bassi et al., 2021; Zhang et al., 2021). Historically, the GBR membrane has aided in stabilizing the blood coagulum and secluding the rapidly proliferating tissue, including the gingival epithelium and connective tissue, thereby creating a suitable environment for osteogenesis (Elgali et al., 2017). Collagen, which has proven biocompatibility and excellent tissue integration (Pei et al., 2021), has received much attention regarding its use in GBR membrane construction (Dacache Neto et al., 2020; Kapogianni et al., 2021). Currently, the GBR membrane is designed with a dual-layered structure, including the layer facing the mucosa and the layer facing the bone defect (Li P. et al., 2021). Notably, driven by the requirement for superior osteogenic performance, there is an urgent need for a bone defect–faced layer to induce and promote bone regeneration. However, premature biodegradation, poor space maintenance ability, undesirable mechanical properties, and insufficient osteogenic capacity make the collagen membrane imperfect. Moreover, the layer facing the bone defect should provide a favorable microenvironment for regulating the fate of osteogenic-related cells (Chuang et al., 2019). In addition, a suitably porous structure of the membrane is also required to facilitate the new bone formation, which guarantees a good supplement of nutrients and enables osteogenic-related cells to grow (Zhang et al., 2017).

The mineralized collagen fibril is the fundamental building block of natural bone (Liu et al., 2016b; Yang et al., 2020b). Compared with pure collagen, it is characterized by advanced biomechanical properties, desirable biodegradation, and superior bone regeneration ability (Pereira et al., 2020). Therefore, the fabrication of porous architecture based on mineralized collagen fibril has generated increased interest (Qiu et al., 2015; Li Z. et al., 2021), and the resulting membrane has been demonstrated to enhance GBR (Li J. et al., 2021). At present, the fabrication procedure for a porous mineralized collagen membrane mainly involves two key processes. The porous collagen membrane is first manufactured and then subjected to mineralization (Wang et al., 2018). Notably, with an increased understanding of natural mineralization, amorphous calcium phosphate (ACP) has been extensively and successfully employed to mineralize collagen fibril (Liu et al., 2016a; Yang et al., 2020a). As demonstrated previously, the physicochemical and biological properties of the resulting mineralized collagen fibril were highly similar to those of the bone (Liu et al., 2016b). Therefore, a significant requirement for the construction of porous mineralized collagen membranes is the fabrication of a porous collagen membrane.

To date, the porous collagen membrane is constructed *via* a bottom-up strategy. In brief, the collagen-rich tissues (such as the rat tail and bovine tendon) are dissolved in a collagen molecule solution using acid, salt, or enzyme extraction (Ferraro et al., 2017). Subsequently, the collagen molecules are assembled and organized into porous collagen membranes using various techniques like freeze-drying, pH adjustment, or electrospinning (Marelli et al., 2015; Zhang et al., 2018; Salvatore et al., 2021; Zhou et al., 2021). Unfortunately, several drawbacks exist during the preparation of porous collagen membranes. First, the collagen extraction process is relatively slow. It usually takes several days to even a week, and the resulting collagen has a low yield and purity (Ferraro et al., 2017). Second, the enzyme removes telopeptides and the acid partially breaks interchain cross-linkages of collagen during the extraction. This technique partially impairs the stability of subsequently assembled collagen fibrils and leads to the loss of topographical cues (Wan et al., 2021). Third, the electrospinning process can severely denature collagen (Zeugolis et al., 2008). Additionally, the pore size, collagen fibril orientation, and membrane thickness cannot be precisely controlled during the pH adjustment or freeze-drying process, impairing new bone formation and clinical application. The aforementioned deficiencies can be mainly attributed to the bottom-up strategy. Altering this fabrication strategy may provide a feasible approach for addressing this challenge.

Bioskiving is a sectioning-based fabrication approach based on a top-down strategy (Alberti and Xu, 2013; Ghazanfari et al., 2019). Instead of employing collagen molecules as the raw material, bioskiving directly utilizes the bovine tendon to construct the collagen membrane (Alberti and Xu, 2016). Therefore, the problems associated with collagen extraction can be avoided. Moreover, the thickness of the collagen membrane can be precisely controlled using a cryomicrotome (Alberti et al., 2014). In addition, as the tendon is composed of highly aligned collagen bundles, numerous collagen membranes with unidirectional fibril architecture can be rapidly fabricated through bioskiving (Alberti and Xu, 2013). Interestingly, as shown using previous studies, the collagen fibril orientation in the meniscus was mildly disrupted after gentle sonication treatment (Yusof et al., 2019), while disordered organization and denatured collagen fibrils were observed in the semitendinosus muscle following a high-power sonication process (Chang et al., 2012). Thus, we hypothesized that proper sonication treatment could alter the unidirectional pattern of the collagen membrane into a randomly organized pattern without collagen denaturation, resulting in the production of a porous collagen membrane that was appropriate for mineralization.

**FIGURE 1:**
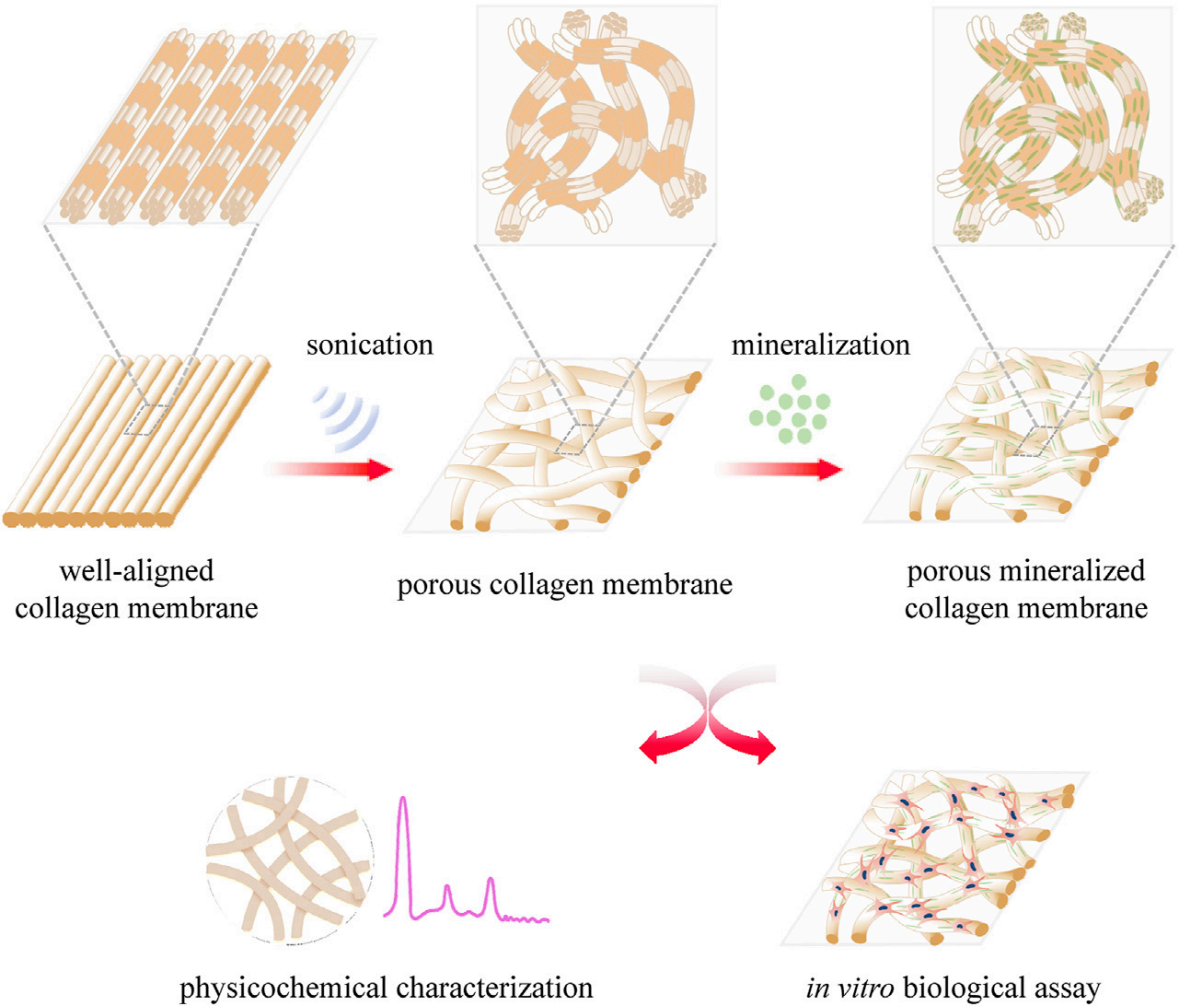
Schematic illustration of the construction of porous collagen membranes *via* bioskiving combined with sonication and mineralization treatment.

The present study describes our attempt to explore a novel method to construct porous collagen membranes *via* a combined bioskiving and sonication treatment approach and verify the feasibility of mineralizing the prepared porous collagen membrane (Figure 1). A series of collagen membranes were first manufactured *via* bioskiving and subsequently sonicated at various power intensities. Using a systematic characterization approach, the influence of sonication power intensity on the physicochemical properties of the collagen membrane was elucidated. Next, porous mineralized collagen membranes were produced using ACP mineralization, and the physicochemical traits (such as microstructure, crystallinity, and inorganic mass) and bioactivity (using preosteoblast cells) were investigated. We expect that from this study, a promising and feasible approach to fabricate the GBR membrane will be developed.

## Materials and Methods

### Fabrication and Characterization of Porous Collagen Membranes

#### Construction of Porous Collagen Membranes *via* Bioskiving and Sonication

Collagen membranes with a unidirectional fibril structure were fabricated through bioskiving (Alberti et al., 2015; Yang et al., 2020a). In brief, the bovine Achilles tendons were purchased from a slaughter house and trimmed into blocks (approximately 10 mm × 10 mm × 2 mm) before immersion in a decellularization solution, containing sodium dodecyl sulfate (1% w/v), ethylenediaminetetraacetic acid (0.1 mM), and Tris buffer (1 mM). The tendon blocks were vigorously shaken in the decellularization solution for 36 h, and the solution was refreshed every 18 h. After a thorough wash with deionized water, the tendon blocks were sectioned into collagen membranes using a cryomicrotome, and the thickness was precisely maintained at 100 μm. Following repeated washes, the bovine tendon membranes were treated at different sonication powers (30, 60, 90, and 120 W) for 20 min using a sonicator (Lifeng Co., China). During the sonication treatment, the collagen membranes were placed on a polytetrafluoroethylene (PTFE) plate and covered with a glass slide to avoid membrane folding. Ice was supplied to the sonic bath to keep the temperature at around 25°C. Next, the prepared porous collagen membranes were cross-linked by 1-ethyl-3-(3-dimethylaminopropyl)-carbodiimide (0.25 M)/N-hydroxysuccinimide (0.1 M) for 2 h. The prepared collagen membranes were labeled according to the power intensity of the sonication treatment: 30 W collagen membrane (WCM), 60, 90, and 120 WCM. All the chemical reagents mentioned before were purchased from Xiya (Xiya Reagent Co., Shangdong, China). In addition, gelatin was used as the control to evaluate whether the sonication treatment denatured the collagen and was obtained by placing bioskiving-fabricated bovine tendon membranes into an 80°C water bath for 20 min.

#### Scanning Electron Microscopy

A SEM (Quanta 400F) was utilized to evaluate the surface and lateral micromorphology of prepared collagen membranes at an accelerating voltage of 15 kV. The 30, 60, 90, and 120 WCMs were dehydrated in a gradient ethanol solution (50–100%), mounted on a stub, and sputter-coated with gold particles. The diameter and orientation of the collagen fibrils were measured and analyzed by ImageJ software.

#### Differential Scanning Calorimetry

DSC (DSC-204 F1) analyses of the 30, 60, 90, and 120 WCMs and gelatin were conducted in a nitrogen atmosphere with temperatures ranging between 100 and 120°C. The heating rate was 5°C/ min.

#### Circular Dichroism

CD measurements were conducted by using a Chirascan Plus spectropolarimeter (Applied Photophysics). The samples were prepared by dissolving the porous collagen membrane into hydrochloric acid (pH = 1), and the concentration was 0.4 mg/ ml. The samples were equilibrated for 1 h before the test, and CD spectra were collected from 190 to 240 nm.

#### Attenuated Total Reflectance–Fourier Transform Infrared Spectroscopy

The functional group and integrity of the triple helix of the collagen fibril were investigated by the ATR-FTIR spectrophotometer (Nicolet iN10). The 30, 60, 90, and 120 WCMs were scanned in the range between 400 and 4,000 cm^−1^. The peak ratio of the amide band III to 1,448 cm^−1^ was calculated to evaluate the integrity of the triple helix.

#### Atomic Force Microscope

The elasticity modulus of the untreated membrane, 30, 60, 90, and 120 WCMs, and gelatin were characterized by using an AFM (MFP-3D-S). The AFM was tested in an AC mode (tapping mode) at room temperature and atmospheric pressure.

#### Surface Wettability Characterization

The water contact angles of membranes were measured by using a contact angle goniometer (Model JY-82). The untreated membrane, 30, 60, 90, and 120 WCMs, and gelatin were stuck to the glass slide. Water droplets dripped at a rate of 2.00 μL/ s onto the membranes.

### Fabrication and Characterization of Porous Mineralized Collagen Membranes

#### Construction of Porous Collagen Membranes

A mineralized solution was prepared based on the previously reported method (Yang et al., 2016; Yang et al., 2020b). One gram of carboxymethyl chitosan (CMCS, Xiya Reagent Co.) was dissolved in Tris buffer containing 2,742 mg of K_2_HPO_4_, followed by addition of (drop-by-drop) 2000 mg of CaCl_2_. The porous collagen membranes were immersed in the mineralized solution and shaken (60 rpm) at room temperature, and the mineralized solution was refreshed every 2 days. After 8 days, the porous mineralized collagen membranes were collected and named according to the power intensity of sonication: 30, 60, 90, and 120 WMCM. Mineralized collagen membranes were washed with deionized water to eliminate the loosely attached minerals and freeze-dried before the physicochemical characterization.

#### SEM and Element Analysis

SEM was employed to investigate the microstructure of mineralized collagen membranes at an accelerating voltage of 15 kV. The samples were prepared according to the aforementioned method. The chemical elements were detected by the energy-dispersive spectrum (EDS).

#### Transmission Electron Microscopy

TEM (FEI Tecnai G2 Spirit) was used to evaluate the intra- and extrafibrillar mineralization of the 30, 60, 90, and 120 WMCMs. Sample powders were prepared by pulverizing porous mineralized collagen membranes in liquid nitrogen and dispersed in ethanol before dropping onto the 300-mesh copper grid.

#### X-Ray Diffractometer and Thermogravimetric Analysis

The crystal structure of 30, 60, 90, and 120 WMCMs was determined by XRD (Empyrean), which was operated at 40 kV and 35 mA. The mineral content of the porous mineralized collagen membranes was investigated using a TGA (TG 209 F1 Libra) under an air atmosphere. The TGA was conducted at a temperature ranging from room temperature to 800°C at a heating rate of 10°C/ min.

### *In Vitro* Biological Assays

#### Cell Culture and Proliferation Assay

MC3T3-E1 preosteoblasts were purchased from Procell Life Science and Technology Co., Ltd (Wuhan, China). The cells were cultured in an alpha modified Eagle’s medium (αMEM, Gibco, NY, United States) in the presence of 10% fetal bovine serum (FBS, Gibco) and 1% penicillin/streptomycin (Gibco) at 37°C and 5% CO_2_. The culture medium was refreshed every 2 days. As cells reached approximately 90% confluence, the MC3T3-E1 cells were passaged at a ratio of 1:3 or seeded onto membranes to conduct biological assays. Both collagen membranes (30, 60, 90, and 120 WCM) and mineralized collagen membranes (30, 60, 90, and 120 WMCM) were cut into 6-mm × 6-mm sections before placing in 96-well plates. The membranes were sterilized with 75% ethanol for 1 day and thoroughly washed with PBS.

As for the cell proliferation assay, MC3T3-E1 cells were seeded on the membrane surface (at a density of 1 × 10^4^) with 100 μL of culture medium. The culture medium was changed every 2 days. On days 1, 3, and 5, cell proliferation was evaluated using the CCK-8 assay kit (Dojindo, Kumamoto, Japan). The culture medium was removed at the determined time, and the membranes were gently rinsed three times with PBS. Subsequently, 90 μL culture medium supplied with 10 μL of CCK-8 reagent was added and incubated for 1 h. Next, the medium was removed, and the optical density (OD) value at 450 nm was observed using the plate reader (Beckman Coulter AD 340). Live/dead cell double staining was performed using the Live/Dead kit (BestBio Science, Shanghai, China) according to the manufacturer’s instructions to investigate the morphology and distribution of cells that grew on the membrane. On day 5, the culture medium was aspirated entirely, and the membrane was seeded with MC3T3-E1 cells and carefully washed with PBS. Subsequently, the membranes were subjected to the calcein-AM staining for 30 min, washed with PBS, stained with PI for 30 min, and last washed with PBS. Finally, the membranes were observed using a fluorescence microscope (Leica DM2500 LED). The percentage of live cells and the cell cover area were measured by ImageJ software.

### Osteogenic Differentiation Assay

Membranes were cut into 8-mm × 8-mm sections and placed in 48-well plates, followed by ethanol (75%) sterilization and PBS washing. MC3T3-E1 cells were seeded at a density of 2 × 10^4^ cells per membrane with 200 μL culture medium. After 2 days, the osteogenic culture medium (MC3T3-E1 culture medium supplemented with 50 μg/ ml ascorbic acid, 10 mM β-glycerol phosphate, and 10 nM dexamethasone) was added and refreshed every 2 days. On day 7, ALP staining was performed. The membranes seeded with cells were rinsed with PBS, fixed by 4% paraformaldehyde, and stained with a BCIP/NBT Alkaline Phosphatase Color Development Kit (Beyotime, Shanghai, China) for 2 h. Then membranes were observed using a stereomicroscope (Leica S9i). Meanwhile, ALP activity was tested on days 7 and 14. The cells that grew on the membranes were washed three times with PBS and then 100 μL of lysate (Beyotime) was added and kept on ice for 1 h . The ALP activity was further tested using the Alkaline Phosphatase Assay Kit (Beyotime). The results were normalized to the total intracellular protein content determined by the Bicinchoninic Acid Protein Assay Kit (Beyotime). The culture medium was collected on days 14 and 21, and OCN levels were measured using the Osteocalcin ELISA kit (Shanghai Enzyme-linked Biotechnology Co., Ltd, Shanghai, China).

### Statistical Analysis

All the experiments were repeated in triplicate. Data are expressed as mean ± standard deviation. One-way ANOVA and a post hoc Bonferroni t-test were employed to evaluate statistical significance. The significance level was set as *p* < 0.05.

## Results

### Synthesis and Characterization of Porous Collagen Membranes

**FIGURE 2:**
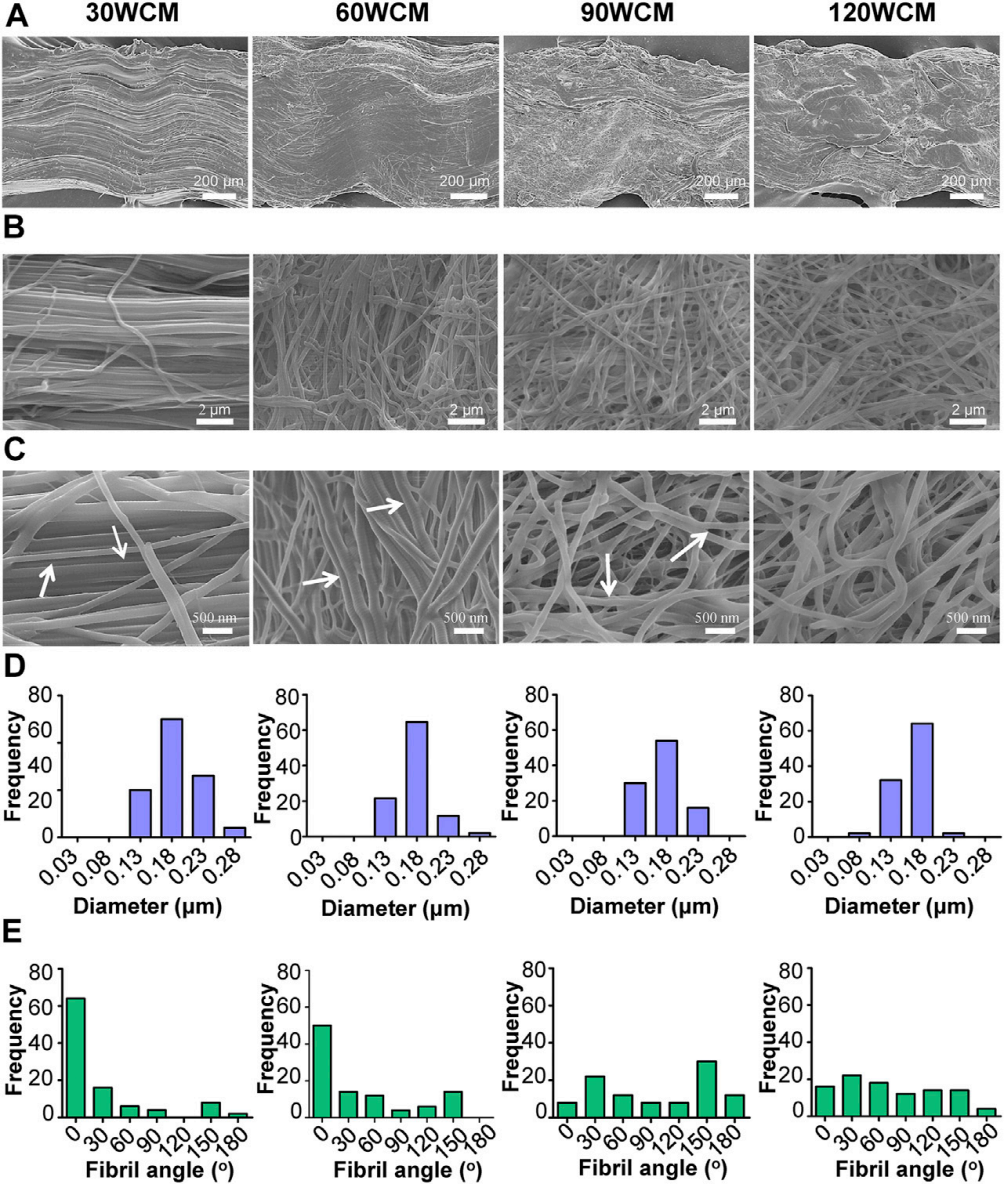
Microstructures of prepared collagen membranes. **(A)** SEM images of lateral view. **(B)** Low-magnification SEM images of surface **(C)** High-magnification SEM images of surface (the arrows represent the cross-band structure of collagen fibrils). Corresponding **(D)** fibril diameter distributions and **(E)** fibril orientation distributions of 30, 60, 90, and 120 WCM.

The microstructure of prepared collagen membranes was investigated by SEM (Figure 2). As the sonic power increased, the lateral section view of the collagen membrane changed from the tightly packed orientated pattern to the loosely packed disordered pattern, indicating that fiber orientations inside the membrane could be altered following the sonication treatment (Figure 2A). Compared with the well-aligned fibril structure of the untreated bovine tendon membrane (Supplementary Figure S1B), only a mild alteration of fibril orientation was observed in the 30 WCM group, and a majority of the fibrils still had a unidirectional organization (Figure 2B). Additionally, the periodic cross-band structure was not affected (Figure 2C). As the sonic power was increased to 60 W, the pattern of parallel alignment was disrupted to some extent (Figure 2E), and several micropores were detected. The cross-band structure was also distinctly found in the collagen fibrils. Notably, the alignment of collagen fibrils was disrupted entirely in the 90 WCM. Numerous micropores emerged in this collagen network, and the cross-band structure could also be found in the fibrils. In the 120 WCM group, the collagen membrane still exhibited a porous architecture combined with disordered organization patterns, and the fibril diameter (approximately 0.18 μm) was comparable to that of the 30, 60, and 90 WCM groups (Figure 2D). However, the cross-band structure became obscure, which was almost similar to the gelatin group (Supplementary Figure S1C).

**FIGURE 3:**
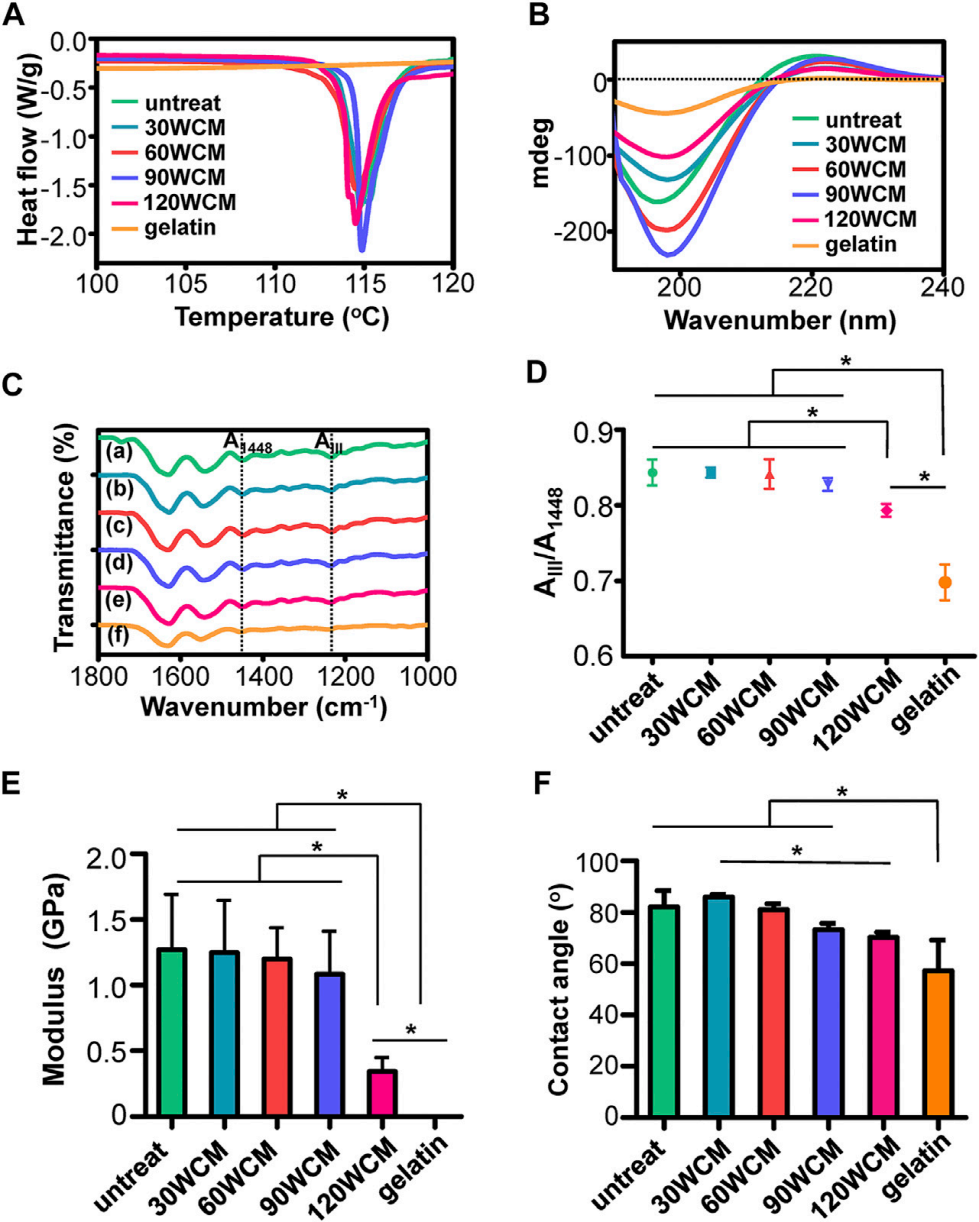
**(A)** DSC and **(B)** CD results of prepared collagen membranes. **(C)** ATR-FTIR spectra, the dotted lines in the ATR-FTIR spectra indicated the peaks at 1,448 cm^−1^ and amide band III, respectively. **(D)** Peak ratio of A_III_/A_1448_ **(A)** untreated collagen membrane, **(B–E)** 30, 60, 90, and 120 WCM, respectively, and **(F)** gelatin. **(E)** Young’s modulus of the prepared collagen membranes. **(F)** The contact angles of water on prepared collagen membranes. The asterisk on top of the bar indicated a statistically significant difference between groups (*p* < 0.05).

The thermal stability of prepared collagen membranes was tested by DSC (Figure 3A). Contrary to the gelatin group, the endothermic peak, associated with the denaturation from a triple helix to the random coil of collagen (He et al., 2011), could be found in the sonication-treated collagen membranes. Figure 3B demonstrates the results of the CD spectra. The gelatin exhibited a single negative peak of lower molar ellipticity, revealing the random conformation of collagen α-chains (Zeugolis et al., 2008). Conversely, the characteristic sinusoidal spectra could be found in the sonication-treated collagen membranes. Notably, the peak intensity of both negative and positive peaks was reduced in the 120 WCM group compared with other groups. To further investigate the influence of sonication power on the collagen fibril structure, ATR-FTIR was conducted to assess the integrity of the triple helix (Figure 3C); (He et al., 2011; Marelli et al., 2015). Three typical amide bands associated with collagen could be detected in the untreated bovine tendon membrane. The amide I band at 1,631 cm^−1^ was assigned to the C=O stretching vibration. The amide II band at 1,541 cm^−1^ was related to the N–H bending vibration coupled with the C–N stretching vibration. The amide III bands at 1,238 cm^−1^ were attributed to N–H bending vibration and C–N stretching. The position and intensity of these amide bands were maintained in the 30, 60, and 90 WCM groups. As reported, the peak ratio of amide band III to 1,448 cm^−1^ was a crucial parameter used to evaluate the integrity of the triple helix (He et al., 2011). Figure 3D shows no significant difference among the untreated, 30, 60, and 90 WCM groups, indicating that sonic power (30, 60, and 90 W) could tune the fibril orientation without destroying the triple helix. Although the peak ratio of the 120 WCM group was significantly higher than that of gelatin, this peak ratio was remarkably lower than that of other sonication-treated collagen membranes. The triple helix of the collagen fibril was partially damaged following the 120 W sonication treatment, and this was confirmed using an AFM. Figure 3E shows that Young’s modulus of the 30, 60, and 90 WCM groups was similar to that of the untreated bovine tendon membrane (approximately 1.2 ± 0.38 GPa). Nevertheless, Young’s modulus of the 120 WCM was dramatically reduced to 0.34 ± 0.10 GPa, while Young’s modulus of gelatin only reached 0.001 ± 0.0002 GPa. All the prepared collagen membranes exhibited a hydrophilic interface. As the sonic power increased, the water contact angle of prepared collagen membranes gradually decreased (Figure 3F).

### Synthesis and Characterization of Porous Mineralized Collagen Membranes

**FIGURE 4:**
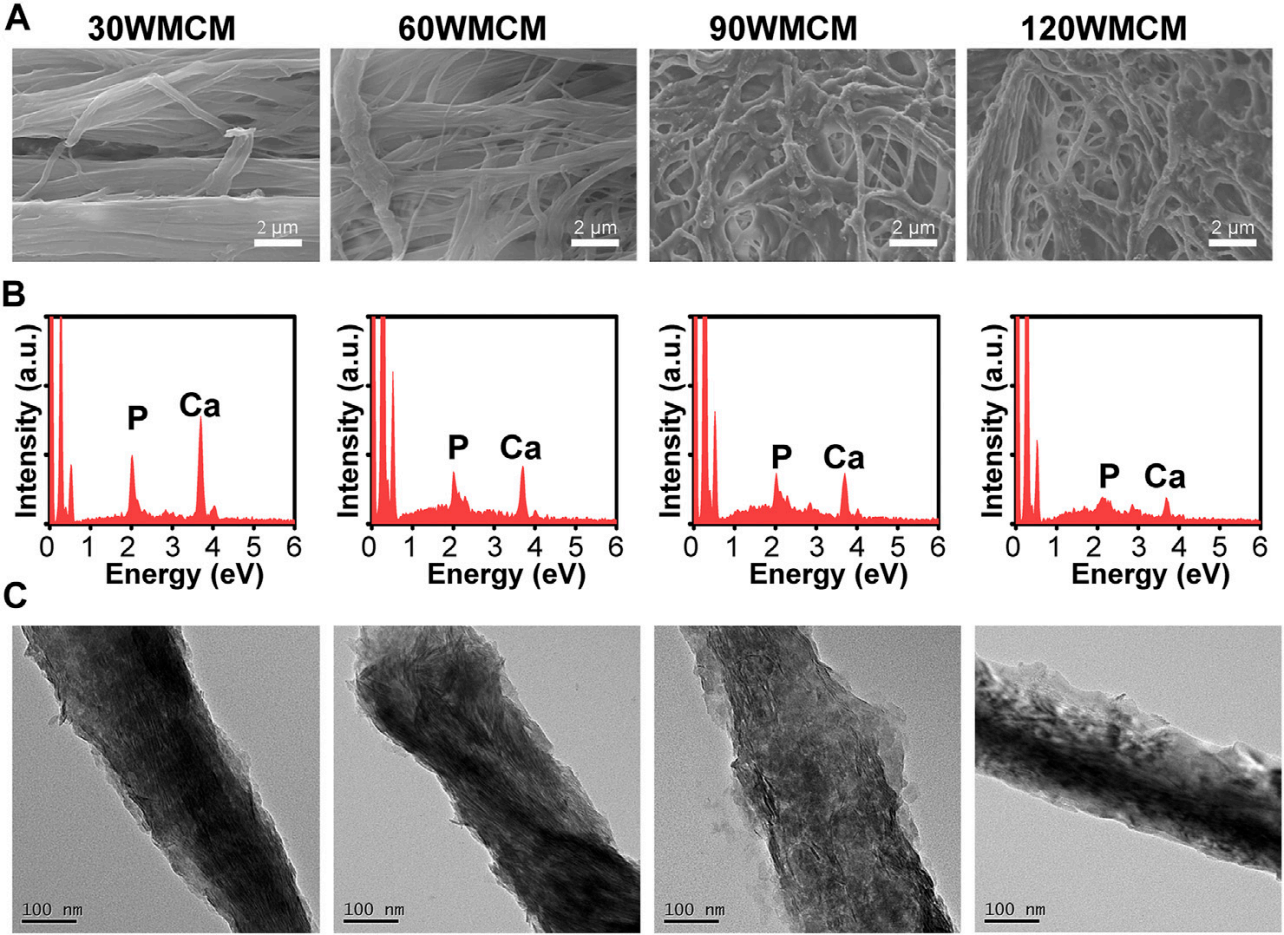
**(A)** SEM images, **(B)** EDS, and **(C)** TEM images of the prepared mineralized collagen membranes.

The micromorphology of mineralized collagen membranes was characterized by SEM (Figure 4A). Compared with an unmineralized collagen membrane, the pattern of fibril organization was not tremendously altered. The 90 and 120 WMCMs still had a porous network architecture. After mineralization, the periodic cross-band structure almost disappeared, and a rougher collagen fibril surface was obtained. The EDS analyses (Figure 4B) verified that these membranes were rich in calcium and phosphate elements, showing that calcium and phosphate were efficiently deposited in the collagen fibril following mineralization. Furthermore, TEM images displayed numerous plate-shaped minerals, intrafibrillarly and extrafibrillarly distributed along the collagen fibril axis in the 30, 60, and 90 WMCM groups. However, minerals were also present in the 120 WMCM group, and the micromorphology of the fibrils was quite different. The fibril contours were indistinct, and this may be ascribed to the partial denaturing of collagen fibrils because of the potent sonication treatment.

**FIGURE 5:**
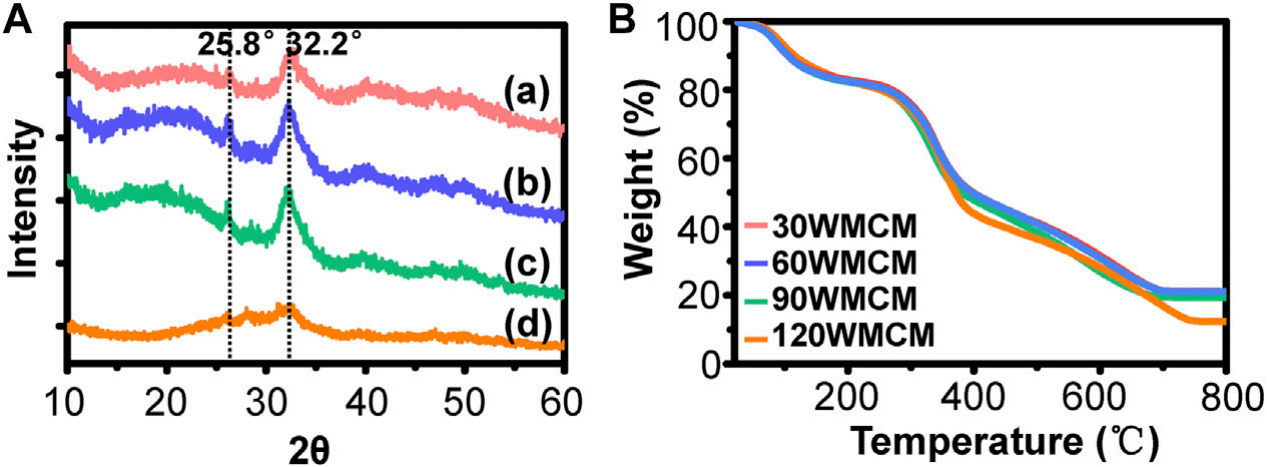
**(A)** XRD and **(B)** TGA results of the prepared mineralized collagen membranes. (a–d) 30, 60, 90, and 120 WMCM, respectively.

To determine the crystallinity of the mineral, an XRD analysis was conducted. Figure 5A shows that the typical peaks of hydroxyapatite (32.2° and 25.8°) were detected in the 30, 60, and 90 WMCM groups, suggesting that the minerals deposited inside the collagen fibril were hydroxyapatite and the constructed mineralized collagen fibril was very similar to the bone tissue. The relevant peaks could also be found in the 120 WMCM group. However, the peak intensity was significantly diminished, revealing that the damaged collagen fibrils may not provide sufficient nucleation sites. The inorganic content of the mineralized collagen membranes was determined by TGA (Figure 5B). This analysis found that the mineral mass of the 30, 60, and 90 WMCM was 21.10, 21.03, and 19.35%, respectively, whereas the 120 WMCM contained a mineral mass of 12.37%.

### *In Vitro* Biological Assay

**FIGURE 6:**
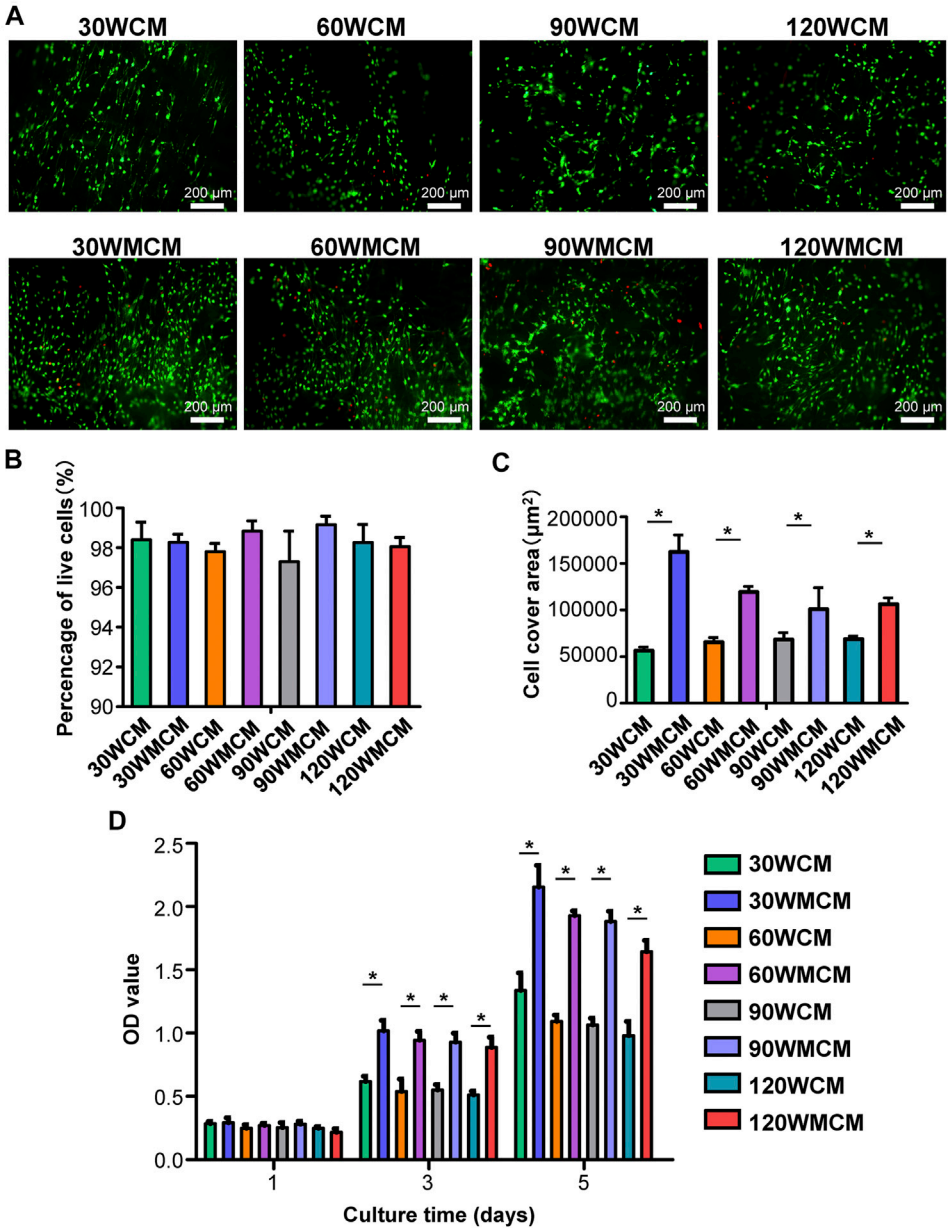
**(A)** Representative live/dead images of cells cultured for 5 days on the unmineralized and mineralized collagen membranes. The live cells were stained green and dead cells were stained red. **(B)** The percentage of live cells and **(C)** cell cover area calculated from live/dead images of cells. **(D)** The CCK-8 results of cell proliferation on days 1, 3, and 5. The asterisk on top of the bar indicates a statistically significant difference between groups (*p* < 0.05).

To monitor the distribution and viability of cells seeded on the membranes, live/dead cell double staining was conducted (Figure 6A). Most cells were viable, and only a few dead cells were observed. The percentage of live cells on each membrane was higher than 90% of all the cells (Figure 6B), demonstrating that all the membranes exhibited a desirable cytocompatibility. Meanwhile, the preosteoblasts spread, and growth was linked to the fibril orientation. As the sonication power increased from 30 to 120 W, the cell arrangement changed from almost unidirectional to random distribution. Notably, the cell density and cell cover area (Figure 6C) were significantly higher in mineralized collagen membranes than in unmineralized collagen membranes. The proliferation of preosteoblasts on membranes is shown in Figure 6D. The number of preosteoblasts from each group increased as the culture time prolonged, demonstrating that the constructed membranes allowed for cell proliferation. On days 3 and 5, the number of preosteoblasts on mineralized collagen membranes was significantly higher than unmineralized collagen membranes, which was consistent with the live/dead cell double staining images.

**FIGURE 7:**
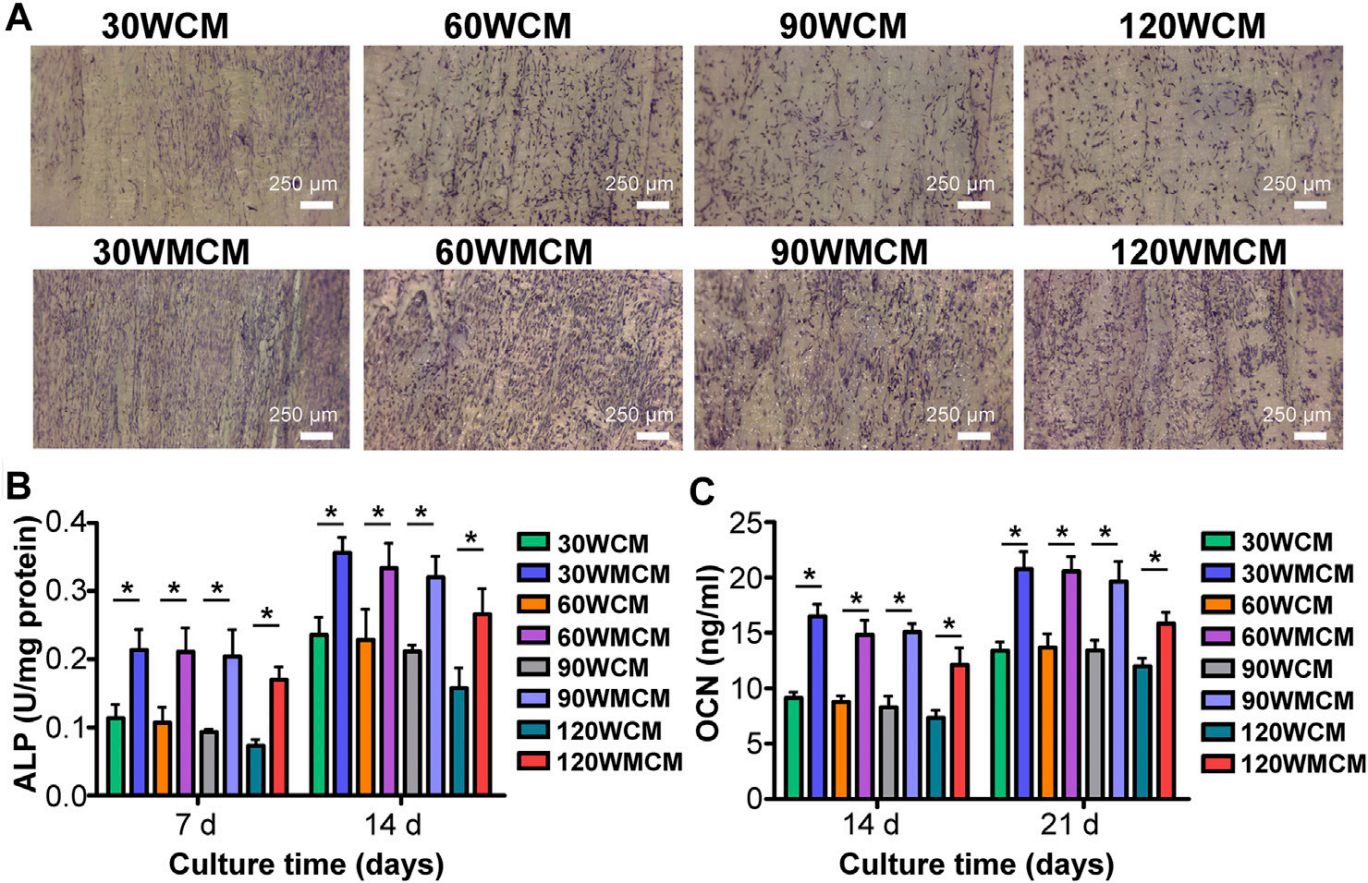
**(A)** Representative images (on day 7) of ALP staining of cells cultured on the unmineralized and mineralized collagen membranes. **(B)** ALP activity and **(C)** OCN content of cells. The asterisk on top of the bar indicated a statistically significant difference between groups (*p* < 0.05).

To analyze the osteogenic differentiation, ALP staining was conducted. Figure 7A shows that the ALP expression was significantly enhanced in mineralized collagen membranes compared with unmineralized collagen membranes, revealing that mineralization improved the osteoinduction ability of membranes. Additionally, similar to the live/dead cell double staining, we observed that the cells were arranged in the direction of the fibril orientation. To further qualitatively compare the osteogenic differentiation ability of mineralized and unmineralized collagen membranes, ALP activity and OCN content were measured. Similarly, we observed that mineralized collagen membranes could significantly enhance the ALP activity and promote OCN expression at predetermined time intervals (Figures 7B,C).

## Discussion

A disordered architecture of type I collagen, for example, the collagen matrix of the woven bone and the cambium layer of the periosteum, was widely found in humans (Reznikov et al., 2014; Chen et al., 2015). Constructing the disordered architecture of collagen is vitally essential for engineering the biomimetic organ. Type I collagen is abundant in humans, and the triple helix is the primary structure of type I collagen. Under the packing arrangement rule, the triple helices are self-assembled into collagen fibrils that have a periodically banded structure, containing alternating overlap and gap zones (Reznikov et al., 2014; Luo et al., 2021). These topological features are vital for collagen fibrils to give biological cues to cells and provide the templates for mineralization (Liu et al., 2016a). However, the current porous collagen membrane is fabricated using the bottom-up strategy that involves collagen extraction and assembly. Both procedures can damage the topological features of the collagen fibril (Alberti and Xu, 2013). To address this, the top-down strategy, which involves the bioskiving approach, can be used to fabricate the collagen membrane with a well-aligned fibril morphology that avoids potential topological damage to the collagen fibril. The thorough decellularization process could remove the majority of original resident cells, and the collagen membranes fabricated by bioskiving exhibited excellent bioactivities without immunological reaction *in vivo* (Alberti et al., 2014; Alberti et al., 2016). After implantation, the collagen membranes can be degraded by collagenase-1, which belonged to the matrix metalloproteinase family and primarily secreted by fibroblasts, neutrophils, monocytes, macrophages, and endothelial cells. During the tissue healing and under the regulation of growth factors, cytokines, and hormones, the expression of collagenase-1 was enhanced and the collagen membranes were gradually degraded (Alberti and Xu, 2016; Helling et al., 2017). In addition, the acid environment under the inflammation condition could also contribute to the degradation of the collagen membrane. Therefore, the collagen membranes fabricated by bioskiving can be eventually replaced by the regenerated tissue. An increased alteration of this well-aligned fibril organization into a randomly distributed orientation without damaging the collagen fibril topology represents a feasible approach to manufacture the porous collagen membrane. Inspired by the previous study that demonstrated that sonication could change the cellulose pattern arrangement (Wang and Cheng, 2009), the power intensity of sonication was shown to be the crucial factor regulating the collagen fibril orientation. Therefore, we combined bioskiving and sonication treatment to fabricate the porous collagen membrane and investigated the effect of power intensity on the physicochemical properties of the collagen membrane.

As the power intensity of sonication increased from 30 to 90 W, the alignment pattern of the collagen fibrils was gradually disrupted, and micropores were also detected in the collagen membrane (Figure 2B). The average diameter of the collagen fibril was not significantly altered, whereas the fibril angle was dramatically changed (Figures 2D,E). As the power intensity reached 120 W, the collagen membrane still had a disordered pattern, and the fibril angle remained unchanged compared with the 90 W collagen membrane. Based on the previous results, we verified that tuning the power intensity of sonication could regulate the fibril orientation of the collagen membrane, and the porous structure could be rapidly fabricated following a 20-min sonication treatment at 90 W. Notably, high-intensity sonication denatured and destroyed the collagen tissue (Cheng et al., 2010). Therefore, the topological integrity of the collagen fibril was further evaluated. Figure 2C shows that a periodic cross-band structure was detected in the 30, 60, and 90 W groups, indicating that the collagen fibril’s triple helices were well-preserved. However, the cross-band structure became indistinct in the 120 W group. Similarly, the CD and ATR-FTIR results also indicated that the triple helix of the 120 WCM was somewhat damaged (Figures 3B–D). Moreover, Young’s moduli of the 30 W, 60 W, 90 W, and the untreated groups were significantly higher than those of the 120 W group (Figure 3E), demonstrating that the collagen fibril was partially denatured following the 120 W sonication treatment.

The results mentioned before prove that the combined application of bioskiving and adequate sonication treatment is a feasible approach for preparing the porous collagen membrane. Recently, synchronous self-assembly/mineralization (SSM) is an emerging novel approach to fabricate mineralized porous collagen membranes (Lin et al., 2019; Liu et al., 2020). In brief, collagen molecules and amorphous mineral nanoparticles stabilized by the polyampholytes were mixed in the acidic solution. As the pH gradually increased, the mineralized collagen fibrils were generated *via* a self-adaptive interaction. Through this approach, both the construction of a disordered architecture and mineralization can be achieved simultaneously. SSM stands as an innovative and convenient avenue to obtaining a disordered structure of mineralized collagen fibril. Its essence however is still based on the bottom-up strategy. The potential collagen denature cannot be avoided, and the orientation of the fibril cannot be precisely controlled *via* SSM. On the contrary, the combined application of bioskiving and sonication treatment, directed by the top-down strategy, could address the aforementioned deficiencies and represent an alternative method to fabricate the mineralized porous collagen membranes. Using this novel approach, the topological structure of the collagen fibril can be highly preserved. Unlike the collagen extraction and assembly procedures that usually take several days to even a week, bioskiving to fabricate collagen membranes can be completed within 2 days. The porous structure is then obtained by the sonication treatment for an additional 20 min. This approach, which features a rapid and straightforward operation, is particularly appropriate for large-scale production. Additionally, this technique does not require the freeze-drying and pH adjustment technique, and the orientation of the collagen fibril can be easily manipulated by regulating the power intensity of sonication. Overall, these adjustments allow for the various collagen membranes to meet diverse needs.

Porous mineralized collagen membranes are perceived to be promising materials that can promote GBR. Therefore, mineralizing prepared porous collagen membranes is an essential aspect of this study. After immersing porous collagen membranes in ACP, the calcium and phosphate elements were detected in all the groups (Figure 4B). Both intra- and extrafibrillar mineralization structures were observed in the 30, 60, and 90 WMCM groups (Figure 4C). This experiment demonstrated that collagen membranes fabricated *via* bioskiving and sonication treatment could be mineralized. The porous mineralized collagen membrane could be obtained by mineralizing the 90 W sonication-treated collagen membrane. Although the 120 WMCM exhibited a porous architecture, its mineralization was impaired. As indicated by TEM, XRD, and TGA results (Figures 4C, 5A,B), the hierarchy of the mineralized fibril was partially damaged, and the inorganic mass was reduced, confirming that the denatured collagen fibril could not provide a template for crystallization. Furthermore, the porous mineralized collagen membrane was shown to efficiently induce the spread and ingrowth of cells (Figure 6). Meanwhile, mineralized collagen membranes significantly promoted the proliferation and osteogenic differentiation of preosteoblasts (Figure 7), which was attributed to the favorable microenvironment (mechanical properties, topographical structures, and released Ca^2+^) provided by mineralized collagen fibrils (Wang et al., 2018). The *in vitro* biological results indicated that our prepared porous mineralized collagen membranes could promote efficient GBR *in vivo*.

## Conclusion

Recognizing the drawbacks of porous collagen membranes manufactured using the bottom-up strategy, the present study explored a novel fabrication approach using the combined application of bioskiving and sonication treatment. Using this method, numerous porous collagen membranes were rapidly and easily manufactured, where the collagen fibril orientation was controlled without damaging its topological structure. These porous collagen membranes were further mineralized *via* ACP, and an enhanced osteogenic performance was observed. In this study, we offered an alternative approach for fabricating the porous mineralized collagen membrane for GBR. This fabrication approach could also be employed to build various fundamental structures of the bone (mimicking bone hierarchy), such as the ordered bone (lamellar bone) and the disordered bone (woven bone).

## Data Availability

The raw data supporting the conclusions of this article will be made available by the authors, without undue reservation.

## References

[B1] Alberti K. A. Xu Q. (2013). Slicing, Stacking and Rolling: Fabrication of Nanostructured Collagen Constructs from Tendon Sections. Adv. Healthc. Mater. 2 (6), 817–821. 10.1002/adhm.201200319 23233344

[B2] Alberti K. A. Xu Q. (2016). Biocompatibility and Degradation of Tendon-Derived Scaffolds. Regen. Biomater. 3 (1), 1–11. 10.1093/rb/rbv023 26816651 PMC4723279

[B3] Alberti K. A. Hopkins A. M. Tang-Schomer M. D. Kaplan D. L. Xu Q. (2014). The Behavior of Neuronal Cells on Tendon-Derived Collagen Sheets as Potential Substrates for Nerve Regeneration. Biomaterials 35 (11), 3551–3557. 10.1016/j.biomaterials.2013.12.082 24461939 PMC3950350

[B4] Alberti K. A. Sun J.-Y. Illeperuma W. R. Suo Z. Xu Q. (2015). Laminar Tendon Composites with Enhanced Mechanical Properties. J. Mater. Sci. 50 (6), 2616–2625. 10.1007/s10853-015-8842-2 25691802 PMC4327911

[B5] Alberti K. A. Neufeld C. I. Wang J. Xu Q. (2016). *In Vivo* Peripheral Nerve Repair Using Tendon-Derived Nerve Guidance Conduits. ACS Biomater. Sci. Eng. 2 (6), 937–945. 10.1021/acsbiomaterials.6b00034 33429503

[B6] Chang H.-J. Xu X.-L. Zhou G.-H. Li C.-B. Huang M. (2012). Effects of Characteristics Changes of Collagen on Meat Physicochemical Properties of Beef Semitendinosus Muscle during Ultrasonic Processing. Food Bioproc. Technol. 5 (1), 285–297. 10.1007/s11947-009-0269-9

[B7] Chen K. Lin X. Zhang Q. Ni J. Li J. Xiao J. (2015). Decellularized Periosteum as a Potential Biologic Scaffold for Bone Tissue Engineering. Acta Biomater. 19, 46–55. 10.1016/j.actbio.2015.02.020 25725472

[B8] Cheng Q. Wang S. Han Q. (2010). Novel Process for Isolating Fibrils from Cellulose Fibers by High-Intensity Ultrasonication. II. Fibril Characterization. J. Appl. Polym. Sci. 115 (5), 2756–2762. 10.1002/app.30160

[B9] Chuang Y.-C. Yu Y. Wei M.-T. Chang C.-C. Ricotta V. Feng K.-C. (2019). Regulating Substrate Mechanics to Achieve Odontogenic Differentiation for Dental Pulp Stem Cells on TiO2 Filled and Unfilled Polyisoprene. Acta Biomater. 89, 60–72. 10.1016/j.actbio.2019.02.040 30836198

[B10] Neto A. M. D. Sartoretto S. C. Duarte I. M. Resende R. F. d. B. Neves Novellino Alves A. T. Mourão C. F. d. A. B. (2020). *In Vivo* Comparative Evaluation of Biocompatibility and Biodegradation of Bovine and Porcine Collagen Membranes. Membranes 10 (12), 423. 10.3390/membranes10120423 PMC7765348 33333940

[B11] Elgali I. Omar O. Dahlin C. Thomsen P. (2017). Guided Bone Regeneration: Materials and Biological Mechanisms Revisited. Eur. J. Oral Sci. 125 (5), 315–337. 10.1111/eos.12364 28833567 PMC5601292

[B12] Bassi A. Bizelli V. Francatti T. Rezende de Moares Ferreira A. Carvalho Pereira J. Al-Sharani H. (2021). Bone Regeneration Assessment of Polycaprolactone Membrane on Critical-Size Defects in Rat Calvaria. Membranes 11 (2), 124. 10.3390/membranes11020124 33572318 PMC7916152

[B13] Ferraro V. Gaillard-Martinie B. Sayd T. Chambon C. Anton M. Santé-Lhoutellier V. (2017). Collagen Type I from Bovine Bone. Effect of Animal Age, Bone Anatomy and Drying Methodology on Extraction Yield, Self-Assembly, thermal Behaviour and Electrokinetic Potential. Int. J. Biol. Macromol. 97, 55–66. 10.1016/j.ijbiomac.2016.12.068 28038914

[B14] Ghazanfari S. Alberti K. A. Xu Q. Khademhosseini A. (2019). Evaluation of an Elastic Decellularized Tendon‐derived Scaffold for the Vascular Tissue Engineering Application. J. Biomed. Mater. Res. 107, 1225–1234. 10.1002/jbm.a.36622 30684384

[B15] He L. Mu C. Shi J. Zhang Q. Shi B. Lin W. (2011). Modification of Collagen with a Natural Cross-Linker, Procyanidin. Int. J. Biol. Macromol. 48 (2), 354–359. 10.1016/j.ijbiomac.2010.12.012 21185325

[B16] Helling A. L. Tsekoura E. K. Biggs M. Bayon Y. Pandit A. Zeugolis D. I. (2017). *In Vitro* Enzymatic Degradation of Tissue Grafts and Collagen Biomaterials by Matrix Metalloproteinases: Improving the Collagenase Assay. ACS Biomater. Sci. Eng. 3 (9), 1922–1932. 10.1021/acsbiomaterials.5b00563 33440550

[B17] Kapogianni E. Alkildani S. Radenkovic M. Xiong X. Krastev R. Stöwe I. (2021). The Early Fragmentation of a Bovine Dermis-Derived Collagen Barrier Membrane Contributes to Transmembraneous Vascularization-A Possible Paradigm Shift for Guided Bone Regeneration. Membranes 11 (3), 185. 10.3390/membranes11030185 33803205 PMC7999168

[B18] Li J. Yan J.-F. Wan Q.-Q. Shen M.-J. Ma Y.-X. Gu J.-T. (2021a). Matrix Stiffening by Self-Mineralizable Guided Bone Regeneration. Acta Biomater. 125, 112–125. 10.1016/j.actbio.2021.02.012 33582360

[B19] Li P. Li Y. Kwok T. Yang T. Liu C. Li W. (2021b). A Bi-layered Membrane with Micro-nano Bioactive Glass for Guided Bone Regeneration. Colloids Surf. B: Biointerfaces 205, 111886. 10.1016/j.colsurfb.2021.111886 34091371

[B20] Li Z. Du T. Ruan C. Niu X. (2021c). Bioinspired Mineralized Collagen Scaffolds for Bone Tissue Engineering. Bioactive Mater. 6 (5), 1491–1511. 10.1016/j.bioactmat.2020.11.004 PMC7680706 33294729

[B21] Lin M. Liu H. Deng J. An R. Shen M. Li Y. (2019). Carboxymethyl Chitosan as a Polyampholyte Mediating Intrafibrillar Mineralization of Collagen *via* Collagen/ACP Self-Assembly. J. Mater. Sci. Technol. 35 (9), 1894–1905. 10.1016/j.jmst.2019.05.010

[B22] Liu Y. Liu S. Luo D. Xue Z. Yang X. Gu L. (2016a). Hierarchically Staggered Nanostructure of Mineralized Collagen as a Bone-Grafting Scaffold. Adv. Mater. 28 (39), 8740–8748. 10.1002/adma.201602628 27530607

[B23] Liu Y. Luo D. Wang T. (2016b). Hierarchical Structures of Bone and Bioinspired Bone Tissue Engineering. Small 12 (34), 4611–4632. 10.1002/smll.201600626 27322951

[B24] Liu H. Lin M. Liu X. Zhang Y. Luo Y. Pang Y. (2020). Doping Bioactive Elements into a Collagen Scaffold Based on Synchronous Self-Assembly/mineralization for Bone Tissue Engineering. Bioact. Mater. 5 (4), 844–858. 10.1016/j.bioactmat.2020.06.005 32637748 PMC7327760

[B25] Luo X. Zhang S. Luo B. Li H. (2021). Engineering Collagen Fiber Templates with Oriented Nanoarchitecture and Concerns on Osteoblast Behaviors. Int. J. Biol. Macromol. 185, 77–86. 10.1016/j.ijbiomac.2021.06.072 34139244

[B26] Marelli B. Ghezzi C. E. Zhang Y. L. Rouiller I. Barralet J. E. Nazhat S. N. (2015). Fibril Formation pH Controls Intrafibrillar Collagen Biomineralization *In Vitro* and *In Vivo* . Biomaterials 37, 252–259. 10.1016/j.biomaterials.2014.10.008 25453955

[B27] Pei Y. Jordan K. E. Xiang N. Parker R. N. Mu X. Zhang L. (2021). Liquid-Exfoliated Mesostructured Collagen from the Bovine Achilles Tendon as Building Blocks of Collagen Membranes. ACS Appl. Mater. Inter. 13 (2), 3186–3198. 10.1021/acsami.0c20330 33398989

[B28] de Melo Pereira D. Eischen-Loges M. Birgani Z. T. Habibovic P. (2020). Proliferation and Osteogenic Differentiation of hMSCs on Biomineralized Collagen. Front. Bioeng. Biotechnol. 8, 554565. 10.3389/fbioe.2020.554565 33195119 PMC7644787

[B29] Qiu Z.-Y. Cui Y. Tao C.-S. Zhang Z.-Q. Tang P.-F. Mao K.-Y. (2015). Mineralized Collagen: Rationale, Current Status, and Clinical Applications. Materials 8 (8), 4733–4750. 10.3390/ma8084733 28793468 PMC5455477

[B30] Reznikov N. Shahar R. Weiner S. (2014). Bone Hierarchical Structure in Three Dimensions. Acta Biomater. 10 (9), 3815–3826. 10.1016/j.actbio.2014.05.024 24914825

[B31] Salvatore L. Gallo N. Natali M. L. Terzi A. Sannino A. Madaghiele M. (2021). Mimicking the Hierarchical Organization of Natural Collagen: Toward the Development of Ideal Scaffolding Material for Tissue Regeneration. Front. Bioeng. Biotechnol. 9, 644595. 10.3389/fbioe.2021.644595 33987173 PMC8112590

[B32] Turri A. Elgali I. Vazirisani F. Johansson A. Emanuelsson L. Dahlin C. (2016). Guided Bone Regeneration Is Promoted by the Molecular Events in the Membrane Compartment. Biomaterials 84, 167–183. 10.1016/j.biomaterials.2016.01.034 26828682

[B33] Wan Y. Gao Y. Shao J. Tumarbekova A. Zhang D. Zhu J. (2021). Effects of Ultrasound and thermal Treatment on the Ultrastructure of Collagen Fibers from Bovine Tendon Using Atomic Force Microscopy. Food Chem. 347, 128985. 10.1016/j.foodchem.2020.128985 33476920

[B34] Wang S. Cheng Q. (2009). A Novel Process to Isolate Fibrils from Cellulose Fibers by High-Intensity Ultrasonication, Part 1: Process Optimization. J. Appl. Polym. Sci. 113 (2), 1270–1275. 10.1002/app.30072

[B35] Wang Y. Hua Y. Zhang Q. Yang J. Li H. Li Y. (2018). Using Biomimetically Mineralized Collagen Membranes with Different Surface Stiffness to Guide Regeneration of Bone Defects. J. Tissue Eng. Regener. Med. 10.1002/term.2670 29691999

[B36] Wu Z. Bao C. Zhou S. Yang T. Wang L. Li M. (2020). The Synergetic Effect of Bioactive Molecule-Loaded Electrospun Core‐shell Fibres for Reconstruction of Critical‐sized Calvarial Bone Defect-The Effect of Synergetic Release on Bone Formation. Cell Prolif 53 (4), e12796. 10.1111/cpr.12796 32202021 PMC7162799

[B37] Yang T. Xiao W. Chen W. Li L. Zhu Z. Sui L. (2016). Effect of Carboxymethyl Chitosan and Aging Time on Synthesis and Storage of Amorphous Calcium Phosphate. j nanosci Nanotechnol. 16 (12), 12582–12589. 10.1166/jnn.2016.12982

[B38] Yang T. Li Y. Hong Y. Chi L. Liu C. Lan Y. (2020a). The Construction of Biomimetic Cementum through a Combination of Bioskiving and Fluorine-Containing Biomineralization. Front. Bioeng. Biotechnol. 8, 341. 10.3389/fbioe.2020.00341 32391345 PMC7193115

[B39] Yang T. Xie P. Wu Z. Liao Y. Chen W. Hao Z. (2020b). The Injectable Woven Bone-like Hydrogel to Perform Alveolar ridge Preservation with Adapted Remodeling Performance after Tooth Extraction. Front. Bioeng. Biotechnol. 8, 11901–11915. 10.3389/fbioe.2020.00119 PMC7047753 32154241

[B40] Yusof F. Sha'ban M. Azhim A. (2019). Development of Decellularized Meniscus Using Closed Sonication Treatment System: Potential Scaffolds for Orthopedics Tissue Engineering Applications. Ijn 14, 5491–5502. 10.2147/ijn.S207270 31410000 PMC6650458

[B41] Zeugolis D. I. Khew S. T. Yew E. S. Y. Ekaputra A. K. Tong Y. W. Yung L.-Y. L. (2008). Electro-spinning of Pure Collagen Nano-Fibres - Just an Expensive Way to Make Gelatin? Biomaterials 29 (15), 2293–2305. 10.1016/j.biomaterials.2008.02.009 18313748

[B42] Zhang J. Ma S. Liu Z. Geng H. Lu X. Zhang X. (2017). Guided Bone Regeneration with Asymmetric Collagen-Chitosan Membranes Containing Aspirin-Loaded Chitosan Nanoparticles. Ijn 12, 8855–8866. 10.2147/ijn.s148179 29276386 PMC5733920

[B43] Zhang L. Yu Y. Feng K.-c. Chuang Y.-c. Zuo X. Zhou Y. (2018). Templated Dentin Formation by Dental Pulp Stem Cells on Banded Collagen Bundles Nucleated on Electrospun Poly (4-vinyl Pyridine) Fibers *In Vitro* . Acta Biomater. 76, 80–88. 10.1016/j.actbio.2018.06.028 29940368

[B44] Zhang W. Li P. Shen G. Mo X. Zhou C. Alexander D. (2021). Appropriately Adapted Properties of Hot-Extruded Zn-0.5Cu-xFe Alloys Aimed for Biodegradable Guided Bone Regeneration Membrane Application. Bioact. Mater. 6 (4), 975–989. 10.1016/j.bioactmat.2020.09.019 33102940 PMC7560602

[B45] Zhou T. Chen S. Ding X. Hu Z. Cen L. Zhang X. (2021). Fabrication and Characterization of Collagen/PVA Dual-Layer Membranes for Periodontal Bone Regeneration. Front. Bioeng. Biotechnol. 9, 630977. 10.3389/fbioe.2021.630977 34178953 PMC8219956

