# Supplementary material for: A Rapid and Convenient Approach to Construct Porous Collagen Membranes via Bioskiving and Sonication-Feasible for Mineralization to Induce Bone Regeneration
Source: Front Bioeng Biotechnol. 2021 Oct 11;9:752506. doi: 10.3389/fbioe.2021.752506 (PMC8542776; doi:10.3389/fbioe.2021.752506)
Supplement: Supplementary file 1 [file Image1.pdf]

Supplementary Material

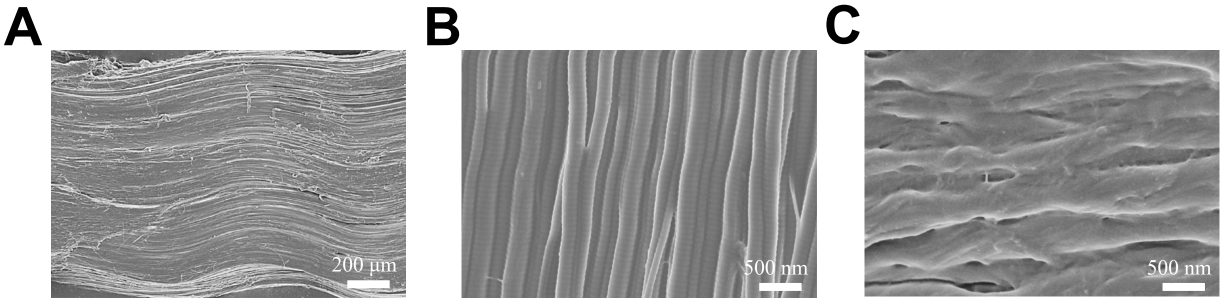

Supplementary Figure 1. The microstructures of collagen membranes. (A) The SEM images of lateral view of untreated collagen membrane. (B) The SEM images of surface view of untreated collagen membrane. (C) The SEM images of surface view of gelatin.
